# Association of *SP1* rs1353058818 and *STAT3* rs1053004 gene polymorphisms with human tongue squamous cell carcinoma

**DOI:** 10.1042/BSR20190955

**Published:** 2019-07-23

**Authors:** Heqing Lai, Guochao Xu, Haifeng Meng, Haiying Zhu

**Affiliations:** 1 Department of Dentistry, Zhejiang Hospital, China; 2 Department of Oral surgery, Hang Zhou Dental Hospital, China; 3 Department of Dentistry, Xiacheng Hospital of Integrated Traditional Chinese and Western Medicine, China

**Keywords:** gene polymorphism, microRNA, tongue squamous cell carcinoma, SP1, STAT3

## Abstract

**Objective:** To study the association between *SP1* rs1353058818 and *STAT3* rs1053004 gene polymorphisms and risk of human tongue squamous cell carcinoma (TSCC).

**Methods:** Sanger sequencing was used to determine the genotypes of *SP1* rs1353058818 and *STAT3* rs1053004 loci in 240 TSCC patients and 240 controls. Levels of hsa-miR-149-5p and hsa-miR-21-5p and expression levels of SP1 and STAT3 proteins in tumor tissues and adjacent normal tissues of TSCC patients were ascertained.

**Results:** Carrying the *SP1* rs1353058818 locus deletion allele was a high risk factor for TSCC (OR = 2.997, 95% CI: 1.389–6.466, *P* = 0.003). The *STAT3* rs1053004 locus A allele was a protective factor for TSCC (OR = 0.604, 95% CI: 0.460-0.793, *P* < 0.001). There was a negative correlation between *SP1* mRNA and hsa-miR-149-5p in tumor and adjacent normal tissues (*r* = −0.81, −0.77). The expression of SP1 protein in tumor tissues of the *SP1* rs1353058818 locus DD genotype was significantly higher than in tissues of the ID type, and in tissues of type II it was the lowest. *STAT3* mRNA was positively correlated with hsa-miR-21-5p in tumor and adjacent normal tissues (*r* = 0.75, 0.78). The expression level of STAT3 protein in tumor tissues of patients with *STAT3* rs1053004 locus GG genotype was significantly higher than in patients with type GA, and it was the lowest in patients with type AA.

**Conclusion:** Polymorphisms in the *SP1* rs1353058818 and *STAT3* rs1053004 loci are associated with the risk of human TSCC.

## Introduction

Tongue squamous cell carcinoma (TSCC) is the most common oral cancer; its incidence is about one-third to half of that of all oral cancers [1]. The pathogenesis of TSCC is related to many factors, including anatomical location, race, age, and sex [2–5]. At present, surgical comprehensive therapy is the main treatment of TSCC, while surgery combined with chemotherapy is employed for advanced TSCC patients. However, due to the side effects of drugs and the problem of distant metastasis, the prognosis is poor [6]. In recent years, gene targeting therapy, which can effectively reduce the toxicity and side effects of drugs, has shown its unique advantages in the clinical treatment of many diseases and has received more and more attention [7,8].

Transcription factor SP1 is one of the main members of the SP protein family. As a sequence-specific DNA-binding protein, it can bind to GC-rich sequences in promoters, thus realizing the regulation of cell and virus gene transcription [9,10]. In normal physiological state, SP1 is widely expressed in various tissues and organs of organism, and has regulatory function for various housekeeping genes. SP1 gene is located in human 12q13.1 region. It contains six exons and encodes 788 amino acid residues. SP1 plays an important role in gene regulation of organisms and in many life processes, such as cell proliferation and apoptosis, differentiation and tumorigenesis [11]. Abnormal expression of SP1 is often associated with the occurrence and progression of tumors. Many studies have shown that the expression of SP1 in gastric cancer [12], pancreatic cancer [13] and breast cancer [14] is significantly higher than that in corresponding normal tissues. Inhibition of SP1 expression could significantly impede cell proliferation and cell cycle progression in TSCC cell lines [15].

Cell transduction and transcription activator 3 (STAT3) is an important member of the signal transduction and transcription activator family. Continuous activation of STAT3 can lead to abnormal cell proliferation and malignant transformation [16,17]. STAT3 gene is located on 17q21.2 gene. The STAT3 protein encoded by STAT3 gene is a component of JAK/STAT signaling pathway, which is an important factor in cell differentiation and cell homeostasis maintenance. The disorder of JAK/STAT signaling pathway is closely related to the occurrence of malignant tumors [18,19]. STAT3 is overexpressed in many malignant tumors, such as breast cancer, head and neck squamous cell carcinoma, colorectal cancer, ovarian cancer, multiple myeloma, leukemia etc. Its expression level is closely related to the growth, differentiation, and metastasis of tumors. Studies have shown that persistent activation of the STAT3 signaling pathway plays an important role in the progression and invasion of human cancers [20,21].

It is still unclear whether polymorphisms of the *SP1* and *STAT3* genes are related to the risk of TSCC. Studies have shown that hsa-mir-149-5p can directly inhibit the expression of SP1 protein to achieve a series of biological processes such as proliferation, invasion, and cell cycle regulation of TSCC cells [22]. In addition, studies have shown that STAT3 mediates the cell survival and cisplatin (DDP) resistance of oral squamous cell carcinoma (OSCC) by up-regulating the expression of hsa-mir-21-5p and down-regulating the downstream targets of hsa-mir-21-5p, including PTEN, TIMP3, and PDCD4 [23]. Therefore, the present study selected deletion mutation sites (rs1353058818) at the binding site of the 3′UTR region of *SP1* gene to hsa-mir-149-5p and of the 3′UTR region of *STAT3* gene to hsa-mir-21-5p (rs1053004) to study the effect of genetic polymorphism on the risk of TSCC.

## Materials and methods

### Patients and test group

Two hundred and forty patients (96 females) from Chinese Han population with TSCC who underwent surgical resection in Zhejiang hospital from February 2015 to February 2018 were included in the present study. They were aged 35–87 years, with an average age of 58.07 ± 12.34 years. Forty-five patients were in TNM (Tumor Node Metastasis) stage I, 63 patients in stage II, 77 patients in stage III, and 55 patients in stage IV. All patients with TSCC were confirmed by pathological examination and had received no treatment before operation. As the control group, 240 healthy volunteers from Chinese Han population were recruited; they were 36–85 years of age, with an average age of 57.45 ± 8.28 years, and 99 were female. The general clinical data of the two groups are compared in Table 1. All the subjects in the present study signed informed consent, which was approved by the Medical Ethics Committee of our hospital.

**Table 1:** General information of the TSCC group and the control group

	TSCC (*n* = 240)	Control (*n* = 240)	*p*
Age (year, mean ± SD)	58.07 ± 12.34	57.45 ± 8.28	0.52
Sex [*n* (%)]			0.78
Male	144 (60.00%)	141 (58.75%)	
Female	96 (40.00%)	99 (41.25%)	
BMI (kg/m^2^, mean ± SD)	26.74 ± 3.53	26.89 ± 2.65	0.60
Smoking [*n* (%)]			0.83
Yes	54 (22.50%)	52 (21.67%)	
No	186 (77.50%)	188 (78.33%)	
Drinking [*n* (%)]			0.43
Yes	73 (30.42%)	81 (33.57%)	
No*	167 (69.58%)	159 (66.25%)	
TNM [*n* (%)]			
I	45 (18.75%)		
II	63 (26.25%)		
III	77 (32.08%)		
IV	55 (22.92%)		

Abbreviations: BMI, body mass index; TNM, tumor node metastasis; TSCC, tongue squamous cell carcinoma.

* Drink no more than three times a year, no more than 50 ml each time.

### Genotyping

The genomic DNA was extracted from 2 ml venous blood of all subjects using the QIAamp® DNA Blood Mini Kit (Qiagen, Hilden, Germany) and stored at −80°C. The primer sequence of rs1353058818 locus was: 5′-GCC TAT TGG GGT TGA GAG GG-3′ (forward); 5′-GCT GCG ACC TTT CTT TCA TCC-3′ (reverse). The primer sequence of rs1053004 locus was: 5′-GCT GAG GCA AGG TGG TTT TG-3′ (forward); 5′-CTG TGC GTA TGG GAA CAC CT-3′ (reverse). The PCR reaction was carried out with 100 ng genomic DNA, 2.5 μl 10× buffer, 1.5 μl Mg^2+^ (25 mM), 0.5 μl dNTP (10 mM), 0.25 μl Taq (5 U/μl), 1 ml forward primer and 1 ml reverse primer, with water added to a total volume of 25 μl. The PCR reaction was carried out as follows: pre-denaturation at 95°C for 5 min, then 30 reaction cycles (denaturation at 94°C for 45 s, annealing at 58°C for 45 s, extension at 72°C for 30 s), and extension at 72°C for 7 min. The PCR products were determined by Sanger sequencing.

### Real-time fluorescence quantitative reverse transcription-polymerase chain reaction (qRT-PCR)

Total RNA was extracted from resected cancer tissues using the RNeasy mini kit (Qiagen, Valencia, CA) kit. A Revert Aid First-Strand cDNA Synthesis kit (Fermentas, Vilnius, Lithuania) was used to synthesize the DNA according to the manufacturer’s instructions. The primer sequences were as follows:

- *SP1*: 5′-CCT GGA TGA GGC ACT TCT GT-3′ (forward); 5′-GCC TGG GCT TCA AGG ATT-3′ (reverse).
- Hsa-mir-149-5p: 5′-AGG GAG GGA CGG GGC UGU GC-3′ (forward); 5′-GTG CAG GGT CCG AGG T-3′ (reverse).
- *STAT3* mRNA: 5′-CCT CTG AGA AGA GGG GAC AA-3′ (forward);5′-CCT CTG AGA AGA GGG GAC AA-3′ (reverse).
- Hsa-miR-21-5p: 5′-GCG CGT CGT GAA GCG TTC-3′ (forward);5′-GCG CGT CGT GAA GCG TTC-3′ (reverse).
- β-Actin: 5′-TGG CAC CAC ACC TTC TAC AAT-3′ (forward); 5′-AGA GGC GTA CAG GGA TAG AGC A-3′ (reverse).

A standard SYBR Green RT-PCR kit (Takara, Dalian, China) was used to carry out qRT-PCR according to the manufacturer’s instructions. The ABI 7500 FAST Real-Time PCR System (Applied Biosystems) was used for the reaction. The reaction conditions were as follows: denaturation at 94°C for 5 min, then 30 reaction cycles (94°C, 30 s; 50°C, 30 s; 72°C, 30 s) and extension at 72°C for 10 min. The levels of *SP1* mRNA, hsa-miR-149-5p, *STAT3* mRNA, and hsa-miR-21-5p relative to β-actin were analyzed by 2^−ΔΔ^*^C^*_t_ and each sample was tested three times.

### Western blotting

Western blotting was used to detect SP1 protein and STAT1 protein in cancer tissues of TSCC patients. SP1 protein antibody (ab157123, Abcam), STAT1 protein antibody (ab157123, Abcam), and 100 ng protein sample were used. All operations were carried out strictly according to the supplier’s instructions.

### Luciferase reporter gene assay

The SP1 3′UTR gene fragment containing hsa-mir-149-5p binding site, including mutant binding site (MT) and wild binding site (WT) fragments, was amplified and then subcloned into pmirGLO vector (Promega, Madison, WI, U.S.A.). To detect luciferase activity, human embryonic kidney (HEK) 293 cells were inoculated into 48-well plates 24 h before transfection. Lipofectamine 2000 was used to co-transfect 10 ng pmirGLO-KLF4 recombinant vector and 50 nM hsa-microRNA-149-5pmimic (5′-UCU GGC UCC GUG UCU UCA CUC CC-3′), hsa-miR-149-5p inhibitor (5′-AGA CCG AGG CAC AGA AGU GAG GG-3′), hsa-miR-149-5p NC (5′-CCA AUG CGU AGG CGG AAU UUA AU-3′) to cells. After 24 h of incubation, according to the manufacturer’s plan, double luciferase assay system (Promega) was used to measure luciferase intensity.

### Statistical analysis

SPSS21.0 (IBM, Chicago, IL, U.S.A.) was used for statistical analysis. The categorized variables were expressed as percentages [*n* (%)] and analyzed with the χ*^2^*test. Hardy–Weinberg equilibrium analysis was also performed by χ*^2^*testing. The means ± SD were used for continuous variables. The association between SNPs and TSCC risk was determined based on the distribution of allele frequencies and genetic models (additive model, dominant model, and recessive model). Odds ratio (OR) and 95% confidence interval (CI) were used in unconditional logistic regression analysis to adjust for age, sex, BMI, smoking, and drinking. Multivariate dimensionality reduction (MDR) was used to analyze the interaction between SNPs at the *SP1* rs1353058818 and *STAT3* rs1053004 loci and age, sex, BMI, smoking, and alcohol consumption. All the tests were double-tailed, and *P* < 0.05 was taken to establish a significant difference.

## Results

### General information

Table 1 summarizes the general clinical information of the 240 TSCC patients and 240 controls. There was no significant difference in age, sex, BMI, smoking, or drinking status between the TSCC and control groups (*P* > 0.05).

### Association of genotype and allele frequencies of SP1 rs1353058818 and STAT3 rs1053004 with TSCC risk

The genotype distribution of *SP1* rs1353058818 and *STAT3* rs1053004 conformed to Hardy–Weinberg equilibrium (*P* > 0.05, Table 2). The risk of TSCC in the *SP1* rs1353058818 dominant model was significantly increased by 2.333 times (95% Cl: 1.040–5.235; *P* = 0.035). Deletion allele carriers were at high risk for TSCC (OR = 2.997; 95% Cl: 1.389–6.466; *P* = 0.003). There was a significant difference in genotype frequencies of *STAT3* rs1053004 between TSCC group and control group (*P* < 0.05). The risk of TSCC was significantly reduced in the dominant and recessive models (*P* < 0.05). The A allele of *STAT3* rs1053004 was the protective factor for TSCC (OR = 0.604; 95% CI: 0.460–0.739; *P* < 0.001).

**Table 2:** Genotype and allele frequencies of *SP1* rs1353058818 and *STAT3* rs1053004

	TSCC (*n* = 240)	Control (*n* = 240)	HWE *P*	Crude OR (95%CI)	*P*	Adjusted OR (95%CI)	*P*
rs1353058818							
II	220 (91.67%)	231 (96.25%)	0.767	1.000 (reference)		1.000 (reference)	
ID	14 (5.83%)	9 (3.75%)		1.871 (0.754–3.942)	0.224	1.633 (0.693–3.850)	0.258
DD	6 (2.50%)	0 (0%)		\		\	
Additive model				1.151 (0.785–1.442)	0.670	1.050 (0.812–1.358)	0.710
Dominant model				2.452 (1.177–7.814)	0.031	2.333 (1.040–5.235)	0.035
Recessive model				\		\	
I	454 (94.58%)	471 (98.13%)		1.000 (reference)		1.000 (reference)	
D	26 (5.42%)	9 (1.88%)		3.124 (1.455–9.548)	0.001	2.997 (1.389–6.466)	0.003
rs1053004							
GG	132 (55.00%)	98 (40.83%)	0.066			1.000 (reference)	
GA	85 (35.42%)	100 (41.67%)		0.655 (0.411–0.953)	0.011	0.631 (0.427–0.932)	0.020
AA	23 (9.58%)	42 (17.50%)		0.448 (0.215–0.788)	0.001	0.407 (0.230–0.720)	0.002
Additive model				0.713 (0.447–1.042)	0.058	0.742 (0.541–1.019)	0.065
Dominant model				0.512 (0.337–0.827)	0.001	0.565 (0.393–0.811)	0.002
Recessive model				0.485 (0.244–0.798)	0.008	0.500 (0.290–0.861)	0.011
G	349 (72.71%)	296 (61.67%)		1.000 (reference)		1.000 (reference)	
A	131 (27.29%)	184 (38.33%)		0.504 (0.412–0.845)	<0.001	0.604 (0.460–0.793)	<0.001

Abbreviations: CI, confidence interval; D, deletion; HWE, Hardy–Weinberg equilibrium; I, insert; OR, odds ratio; TSCC, tongue squamous cell carcinoma.

### Stratified analysis of general clinical information for association of SP1 rs1353058818 gene polymorphism with TSCC risk

We analyzed the association of *SP1* rs1353058818 gene polymorphism with TSCC risk according to age, sex, BMI, smoking, and drinking. The results are shown as Table 3. The risk of TSCC was increased in D allele carriers among subjects with age <60 years old, BMI ≥ 26 kg/m^2^ and who were non-smoking (*p* < 0.05).

**Table 3:** Stratified analysis of general clinical information for association of *SP1* rs1353058818 gene polymorphism with TSCC risk

	TSCC (*n* = 240)	Control (*n* = 240)	Adjusted OR (95%CI)	*P*
Age [year, *n* (%)]				
≥60				
II	93 (87.74%)	42 (100.00%)	1.000 (reference)	
ID/DD	13 (12.26%)	0 (0%)	1.452 (1.023–1.452)	0.017
<60				
II	127 (94.78%)	189 (95.45%)	1.000 (reference)	
ID/DD	7 (5.22%)	9 (4.55%)	1.157 (0.420–3.188)	0.777
Sex [*n* (%)]				
Male				
II	133 (92.36%)	135 (95.74%)	1.000 (reference)	
ID/DD	11 (7.64%)	6 (4.26%)	1.861 (0.669–5.177)	0.228
Female				
II	87 (90.63%)	96 (96.97%)	1.000 (reference)	
ID/DD	9 (9.38%)	3 (3.03%)	3.310 (0.868–12.623)	0.065
BMI (kg/m^2^, mean ± SD)				
≥26				
II	136 (90.07%)	149 (96.75%)	1.000 (reference)	
ID/DD	15 (9.93%)	5 (3.25%)	3.287 (1.164–9.285)	0.018
<26				
II	84 (94.38%)	82 (95.35%)	1.000 (reference)	
ID/DD	5 (5.62%)	4 (4.65%)	1.220 (0.316–4.705)	0.772
Smoking [*n* (%)]				
Yes				
II	52 (96.30%)	48 (92.31%)	1.000 (reference)	
ID/DD	2 (3.70%)	4 (7.69%)	0.462 (0.081–2.635)	0.374
No				
II	168 (90.32%)	183 (97.34%)	1.000 (reference)	
ID/DD	18 (9.68%)	5 (2.66%)	3.921 (1.424–10.796)	0.005
Drinking [*n* (%)]				
Yes				
II	64 (87.67%)	78 (96.30%)	1.000 (reference)	
ID/DD	9 (12.33%)	3 (3.70%)	3.656 (0.950–14.073)	0.090
No				
II	156 (93.41%)	153 (96.23%)	1.000 (reference)	
ID/DD	11 (6.59%)	6 (3.77%)	1.798 (0.649–4.983)	0.253

Abbreviations: CI, confidence interval; D, deletion; I, insert; OR, odds ratio; TSCC, tongue squamous cell carcinoma.

### Stratified analysis of general clinical information for association of STAT3 rs1053004 gene polymorphism with TSCC risk

We analyzed the association of *STAT3* rs1053004 gene polymorphism with TSCC risk by age, sex, BMI, smoking, and drinking. The results are shown in Table 4. The risk of TSCC was decreased in A allele carriers among male subjects with age < 60 years old, BMI ≥ 26 kg/m^2^, who were smokers, and non-drinking (*P* < 0.05, Table 4).

**Table 4:** Stratified analysis of general clinical information for association of *STAT3* rs1053004 gene polymorphism with TSCC risk

	TSCC (*n* = 240)	Control (*n* = 240)	Adjusted OR (95%CI)	*P*
Age [year, *n* (%)]				
≥60				
GG	59 (55.66%)	17 (40.48%)	1.000 (reference)	
GA/AA	47 (44.34%)	25 (59.52%)	0.542 (0.262–1.119)	0.096
<60				
GG	73 (54.48%)	81 (40.91%)	1.000 (reference)	
GA/AA	61 (45.52%)	117 (59.09%)	0.579 (0.372–0.901)	0.015
Sex [*n* (%)]				
Male				
GG	81 (56.25%)	55 (39.01%)	1.000 (reference)	
GA/AA	63 (43.75%)	86 (60.99%)	0.497 (0.310–0.798)	0.004
Female				
GG	51 (53.13%)	43 (43.43%)	1.000 (reference)	
GA/AA	45 (46.88%)	56 (56.57%)	0.678 (0.385–1.191)	0.176
BMI (kg/m^2^, mean ± SD)				
≥26				
GG	81 (53.64%)	67 (43.51%)	1.000 (reference)	
GA/AA	70 (46.36%)	87 (56.49%)	0.666 (0.424–1.045)	0.077
<26				
GG	51 (57.30%)	31 (36.05%)	1.000 (reference)	
GA/AA	38 (42.70%)	55 (63.95%)	0.420 (0.229–0.772)	0.005
Smoking [*n* (%)]				
Yes				
GG	35 (64.81%)	16 (30.77%)	1.000 (reference)	
GA/AA	19 (35.19%)	36 (69.23%)	0.241 (0.107–0.543)	<0.001
No				
GG	97 (52.15%)	82 (43.62%)	1.000 (reference)	
GA/AA	89 (47.85%)	106 (56.38%)	0.710 (0.472–1.067)	0.099
Drinking [*n* (%)]				
Yes				
GG	36 (49.32%)	37 (45.68%)	1.000 (reference)	
GA/AA	37 (50.68%)	44 (54.32%)	0.864 (0.459–1.629)	0.652
No				
GG	96 (57.49%)	61 (38.36%)	1.000 (reference)	
GA/AA	71 (42.51%)	98 (61.64%)	0.460 (0.296–0.717)	0.001

Abbreviations: CI, confidence interval; D, deletion; I, insert; OR, odds ratio; TSCC, tongue squamous cell carcinoma.

### MDR analysis of interaction between SNPs at SP1 rs1353058818 and STAT3 rs1053004 loci and environmental factors

**Figure 1:**
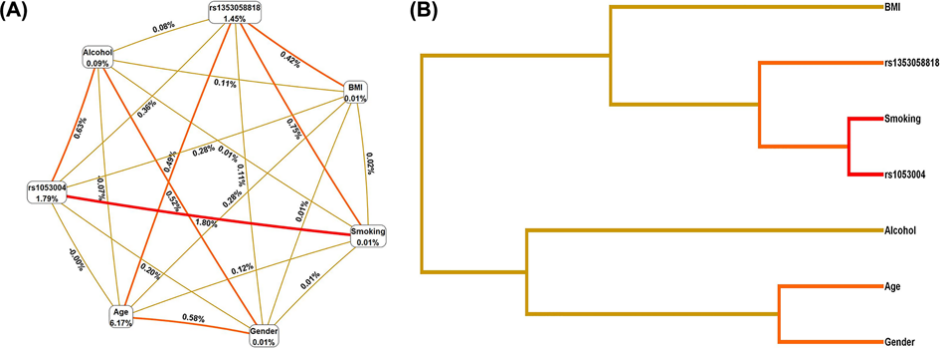
Multifactor dimensionality reduction analysis of interaction between gene and environment (**A**) Ring graph. The data at the vertex represent the contribution to TSCC. The data on the line represent the interaction intensity between the factors at the two vertices. The larger the value, the stronger the interaction. (**B**) Tree diagram. Strong interactions come together and weak interactions stay away from each other.

The interaction between SNPs at the *SP1* rs1353058818 and *STAT3* rs1053004 loci and age, sex, BMI, smoking, and drinking was analyzed by multifactor dimensionality reduction (MDR). The results showed that there was a strong interaction between smoking and SNP at the *STAT3* rs1053004 locus, followed by SNP at the *SP1* rs1353058818 locus (Figure 1). The best predictive model was smoking, rs1053004, rs1353058818, with a prediction error of 0.360 (1-testing balanced accuracy), and the largest cross-validation (10/10, Table 5).

**Table 5:** Best prediction model of the interaction between SNPs at *SP1* rs1353058818 and *STAT3* rs1053004 loci and environmental factors

Model	bal.acc.cv training	bal.acc.cv testing	χ*^2^*	*P*	CV consistency
Smoking	0.633	0.633	3.452	0.214	7/10
Smoking, rs1353058818	0.638	0.638	4.126	0.132	9/10
Smoking, rs1053004, rs1353058818	0.651	0.640	25.142	<0.001	10/10
Smoking, gender, age, rs1053004	0.660	0.598	5.214	0.065	9/10
Smoking, gender, age, rs1353058818, rs1053004	0.677	0.613	4.075	0.106	8/10
Smoking, gender, BMI, age, alcohol, rs1053004	0.693	0.563	9.542	0.002	9/10
Smoking, gender, BMI, age, alcohol, rs1353058818, rs1053004	0.713	0.585	10.256	0.001	10/10

Training Bal. ACC, training balanced accuracy; Testing Bal. ACC, testing balanced accuracy; CV, cross-validation.

### Expression of SP1 mRNA and hsa-miR-149-5p in tumor tissues and normal adjacent tissues

**Figure 2:**
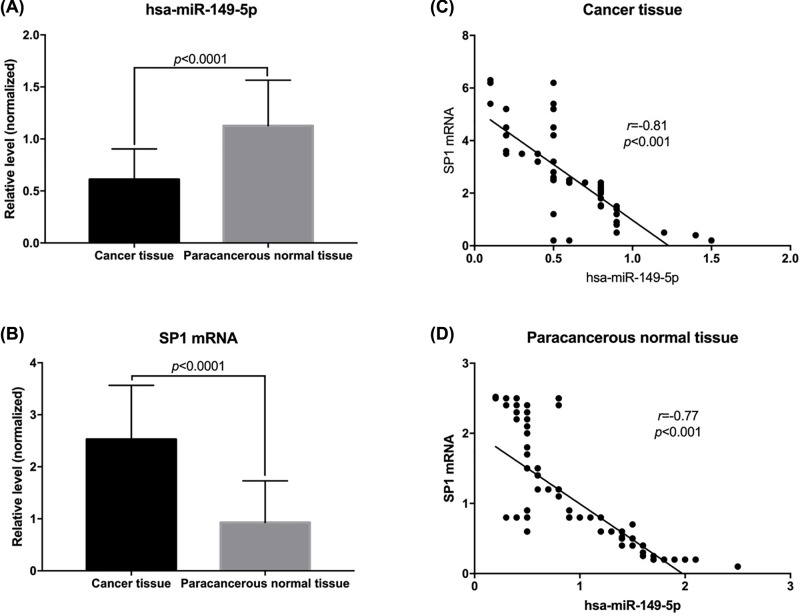
Expression of *SP1* mRNA and hsa-miR-149-5p in tumor tissues and normal adjacent tissues (**A**) Levels of hsa-miR-149-5p in tumor tissues and normal adjacent tissues. (**B**) Levels of *SP1* mRNA in tumor tissues and normal adjacent tissues. (**C**) Correlation between *SP1* mRNA and hsa-miR-149-5p levels in tumor tissues. (**D**) Correlation between *SP1* mRNA and hsa-miR-149-5p levels in normal adjacent tissues.

The levels of *SP1* and hsa-miR-149-5p in resected tumors and adjacent normal tissues were detected by RT-PCR. The results showed that the levels of hsa-miR-149-5p in tumors were significantly lower than those in adjacent normal tissues (*P* < 0.05, Figure 2A), while the levels of *SP1* mRNA in tumors were significantly higher than those in adjacent normal tissues (*P* < 0.05, Figure 2B). Further analysis of the correlation between *SP1* mRNA and hsa-miR-149-5p in cancer tissues and adjacent normal tissues showed that *SP1* mRNA and hsa-miR-149-5p were negatively correlated (*r* = −0.81, −0.77; Figure 2C,D).

### Correlation between SP1 protein expression level and a SNP at SP1 rs1353058818 in tumor tissues

**Figure 3:**
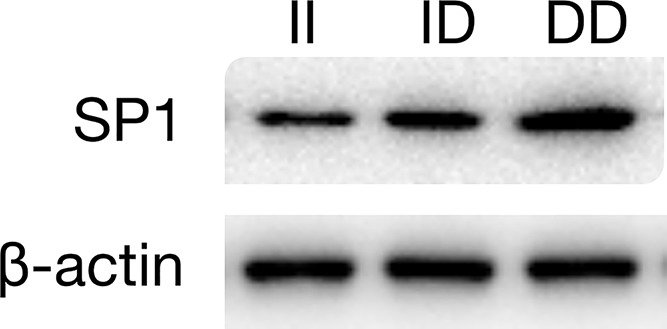
Expression of SP1 protein in tumor tissues of TCSS patients with different genotypes at the *SP1* rs1353058818 locus

Western blots were used to detect the expression of SP1 protein in tumor tissues of TCSS patients. The results showed that the expression of SP1 protein in tumor tissues of DD genotype patients was significantly higher than that of ID genotype patients and that the expression of SP1 protein in tumor tissues of II genotype patients was the lowest, as shown in Figure 3.

### Expression of STAT3 mRNA and hsa-miR-21-5p in tumor tissues and normal adjacent tissues

**Figure 4:**
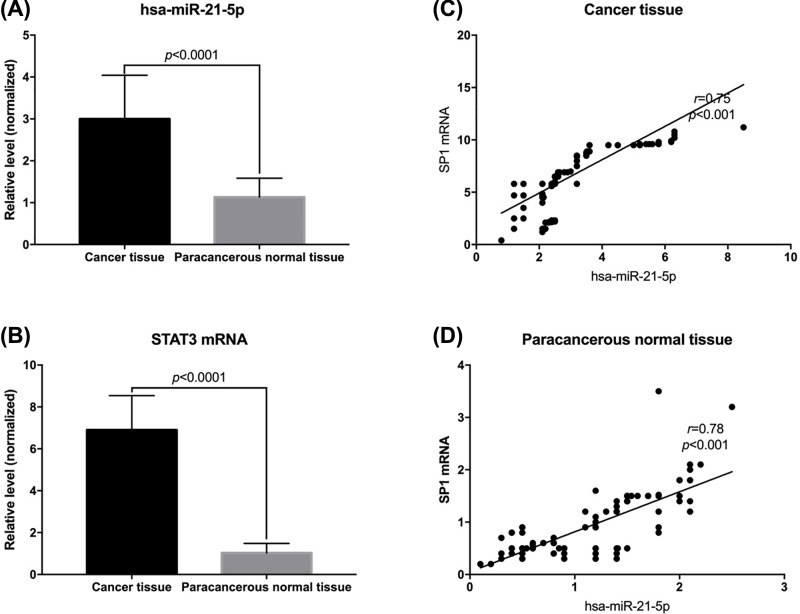
Expression levels of *STAT3* mRNA and hsa-miRNA-21-5p in tumor tissues and adjacent normal tissues (**A**) Levels of hsa-miR-21-5p in tumor tissues and adjacent normal tissues. (**B**) Levels of *STAT3* mRNA in tumor tissues and adjacent normal tissues. (**C**) Correlation between *STAT3* mRNA and hsa-mir-21-5p levels in tumor tissues. (**D**) Correlation between *STAT3* mRNA and hsa-mir-21-5p levels in adjacent normal tissues.

The levels of *STAT3* mRNA and hsa-miR-21-5p in resected tumors and adjacent normal tissues were detected by RT-PCR. The results showed that the levels of *STAT3* mRNA and hsa-mir-21-5p in tumors were significantly higher than those in adjacent normal tissues (*P* < 0.05; Figure 4A,B). Further analysis showed that there was a positive correlation between *STAT3* mRNA and hsa-mir-21-5p in both tumor tissues and adjacent normal tissues (*r* = 0.75, 0.78; Figure 4C,D).

### Correlation between STAT3 protein expression level and SNP of STAT3 rs1053004 locus

**Figure 5:**
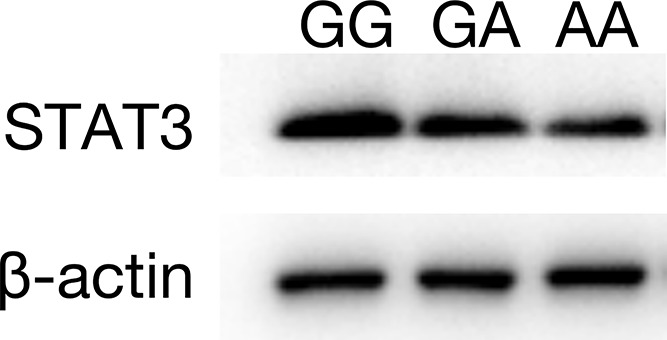
STAT3 protein expression in tumor tissues of TSCC patients with different genotypes at the *STAT3* rs1053004 locus

Western blotting was used to detect the expression of STAT3 protein in tumor tissues of TCSS patients. The results showed that the expression of STAT3 protein in tumor tissues of GG genotype patients was significantly higher than that of GA genotype patients. The expression of STAT3 protein in tumor tissues of AA genotype TSCC patients was the lowest, as shown in Figure 5.

### Target binding of hsa-miR-149-5p and SP1 3′UTR

**Figure 6:**
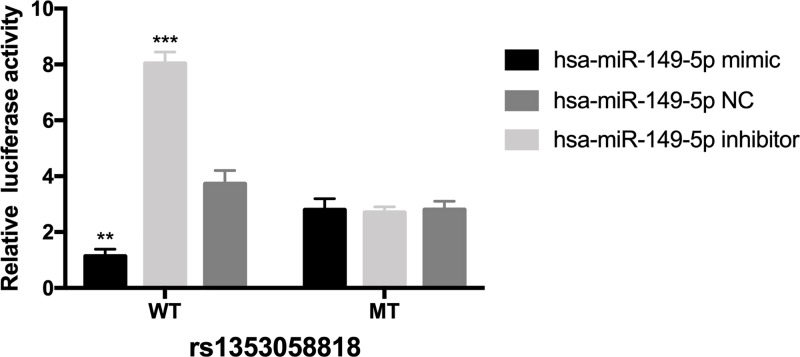
Results of dual luciferase reporter gene assay **P< 0.01, ***P< 0.001.

In order to further prove whether hsa-mir-149-5p can target binding with SP1 3′UTR rs1353058818, we constructed a dual luciferase reporter gene system. SP1 3′UTR region contains 50 bp upstream and downstream fragments of rs1353058818 locus, which are cloned into the downstream of Luciferase reporter gene in PmirGLO to obtain wild-type WT reporter vector. In addition, a mutant MT reporter vector was constructed and the sequence of the reporter vector was demonstrated to be correct. Lipofectamine 2000 was used to co-transfect 10 ng pmirGLO-KLF4 recombinant vector and 50 nM hsa-miR-149-5p NC into cells, respectively. The results showed that the luciferase activity of wild-type WT reporting vector was significantly inhibited after overexpression of hsa-miR-149-5p, while the luciferase activity of mutant MT reporting vector had no significant change (Figure 6).

## Discussion

The incidence of TSCC is related not only to smoking, drinking, obesity, and diet habits, but also to genetic factors [24–26]. Genetic factors associated with TSCC were found to have important clinical significance for early prevention and treatment.

At present, more and more studies have confirmed that miRNA could regulate the expression of SP1. Studies such as Zhu et al. [27] have shown that miR-145 can enhance the sensitivity of ovarian cancer cells to paclitaxel by targeting SP1. Wang et al. [28] showed that SP1 was the target of microRNA-149, and that microRNA-149 could play an anti-cancer role in CRC by inhibiting the expression of SP1. In our study, the expression of hsa-mir-149-5p in TSCC and in TSCC cell lines was significantly reduced, which was consistent with the results of Chen et al. [22]. Their study suggests that the decreased expression of hsa-mir-149-5p is related to the proliferation and invasion of TSCC, and that the role of hsa-mir-149-5p is similar to that of tumor suppressor gene in the deterioration of TSCC. However, there is insufficient evidence to support the specific mechanism of its anti-cancer effect. Therefore, in the present study, using the TargetScan (http://www.targetscan.org/mamm_31/) tool, we predicted that hsa-mir-149-5p could target the 3′UTR region of the *SP1* gene. The binding region was located at nucleotides 3791-3815 of the 3′UTR region. The deletion mutation at the rs1353058818 locus in this region was a risk factor for TSCC, and the risk of TSCC in deleted allele carriers increased 2.997 times. Further analysis showed that there was a negative correlation between *SP1* mRNA and hsa-mir-149-5p in tumor tissues and adjacent normal tissues, suggesting that hsa-mir-149-5p may inhibit the occurrence of TSCC by inhibiting the expression of SP1. By analyzing the expression of SP1 protein in TSCC patients with different genotypes, we found that the expression of SP1 protein in tumor tissues of patients with the deletion mutation was significantly higher than that of patients with the insertion mutation. The conclusion was that the deletion mutation affected the binding of hsa-mir-149-5p to SP1 and its regulation. Interestingly, we found that the risk of TSCC in subjects with D allele was increased only in subjects aged less than 60, BMI > 26 kg/m^2^ and non-smokers (*P* < 0.05), indicating that rs1353058818 SNP interacted with age, BMI, smoking, and other factors.

It has been found that the STAT3 signaling pathway plays an important role in the anti-cancer effect induced by Cryptotanshinone in human TSCC [20]. TargetScan predicted that *STAT3* was the target gene of hsa-mir-21-5p, and some studies found that inhibiting hsa-mir-21-5p could promote the recovery of spinal cord injury by down-regulating the IL-6R/JAK-STAT signaling pathway and inhibiting inflammation [29]. The SNP of the rs1053004 locus is located in the regions of hsa-mir-21-5p and 3′UTR of *STAT3*. In this study, we found that carriers of the rs1053004 locus A allele have a lower risk of TSCC. The binding efficiency of hsa-mir-21-5p to the 3′UTR region of *STAT3* is low in carriers of the A allele, which leads to a decrease of the STAT3 protein expression level. However, the specific mechanism needs further study.

It is interesting to note that only in subjects aged less than 60, male, BMI < 26 kg/m^2^, smokers, and non-drinkers, subjects with the *STAT3* rs1053004 locus and the A allele (GA/AA) had a lower risk of TSCC, indicating that the association between a SNP at the *STAT3* rs1053004 locus and TSCC risk interacted with factors such as age, sex, BMI, smoking, and alcohol consumption. At the same time, the results of MDR analysis showed strong interactions among smoking, SNPs of rs1053004, and SNPs of rs1353058818. Therefore, the interaction between genes and environmental factors has an important impact on the occurrence of TSCC.

The study has some shortcomings. To begin, we lack direct evidence to support that hsa-mir-149-5p regulates the *SP1* and hsa-mir-21-5p regulates *STAT3* genes in the genesis of TSCC. Second, because the sample size is small and the number of patients with these rare mutations is small, the error of analysis may be magnified, and the sample size needs to be further expanded. Nevertheless, the present study is a good attempt to explore the role of genetic factors in gene expression regulated by miRNAs. The process of interaction between multiple genes and factors during the occurrence of TSCC may be complex. Therefore, the selected genes and SNP loci in this study are far from enough.

## Conclusions

The deletion mutation at the *SP1* rs1353058818 locus is a high risk factor for TSCC, and the A allele at the *STAT3* rs1053004 locus is a protective factor for TSCC. The possible reason is that the deletion mutation affects the targeting regulation of hsa-mir-149-5p to the *SP1* gene. The binding efficiency of allele A at the *STAT3* rs1053004 locus to hsa-mir-21-5p is low, which affects the regulation of TSCC. However, further studies are needed to confirm the effect.

## Abbreviations

BMI: body mass index
TNM: tumor node metastasis
TSCC: tongue squamous cell carcinoma
